# Similar Metabolic Changes Induced by HIPVs Exposure as Herbivore in *Ammopiptanthus mongolicus*

**DOI:** 10.1371/journal.pone.0095474

**Published:** 2014-04-18

**Authors:** Jingru Sun, Xiao Zhang, Chuanjian Cao, Xindi Mei, Ningning Wang, Suli Yan, Shixiang Zong, Youqing Luo, Haijun Yang, Yingbai Shen

**Affiliations:** 1 National Engineering Laboratory for Tree Breeding, College of Biological Sciences and Technology, Beijing Forestry University, Beijing, China; 2 Forest Pest control and Quarantine Station of Ningxia, Yinchuan, China; 3 Key Laboratory for Silviculture and Conservation, Ministry of Education, Beijing Forestry University, Beijing, China; 4 Beijing Key Laboratory for Microanalytical Methods and Instrumentation, Department of Chemistry, Tsinghua University, Beijing, China; Pennsylvania State University, United States of America

## Abstract

Herbivore-induced plant volatiles (HIPVs) are important compounds to prim neighboring undamaged plants; however, the mechanism for this priming process remains unclear. To reveal metabolic changes in plants exposed to HIPVs, metabolism of leaves and roots of *Ammopiptanthus mongolicus* seedlings exposed to HIPVs released from conspecific plants infested with larvae of *Orgyia ericae* were analyzed together with control and infested seedlings using nuclear magnetic resonance (NMR)-based metabolic technology and multi variate data analysis. Results presented showed that HIPVs exposure led to similar but specific metabolic changes compared with those induced by infestation in both leaves and roots. Furthermore, both HIPVs exposure and herbivore attack resulted in metabolic changes involving a series of primary and secondary metabolites in both leaves and roots. Taken together, these results suggested that priming of yet-damaged plants may be achieved by reconfiguring metabolic pathways in leaves and roots to make similar concentrations for all metabolites as those in seedlings infested. Therefore, we propose that improved readiness of defense induction of primed plants toward subsequent herbivore attack may be based on the similar metabolic profiling induced by HIPVs exposure as those caused by herbivore.

## Introduction

Over several hundred million years of battle between plants and herbivores [1], plants are documented to have evolved various strategies to adapt to or counteract herbivores [2]. Among these strategies, releasing of herbivore-induced plant volatiles (HIPVs) upon attack is an important way for plants to defend herbivorous insects. HIPVs are volatile organic compounds, mainly including products of shikimic acid-pathway, fatty acid-derived products and terpenes. One main function of HIPVs is the ability to prim neighboring undamaged plants. Plants primed by HIPVs increase readiness of defense induction [3] and respond to subsequent herbivores actively. To reveal the mechanism for the earlier or stronger response for primed plants in response to subsequent herbivore, many studies focus on the changes of plants exposed to HIPVs.

Experiments under laboratory conditions and field indicate that plants exposed to HIPVs show defensive characters. To reveal the mechanism of HIPVs induced priming, studied focused on HIPVs induced changes in plants. Higher level of jasmonic acid [4],increased expression of some defense-related genes [5], [6], [7], [8], differences in quantities and qualities of volatiles organic compounds [9], induced extrafloral nectar secretion [10], and reduced larval development [11] were observed in plants exposed to HIPVs. However, integral metabolic changes of leaves and crosstalk between above- and below ground parts of HIPVs-receiver plants are quite poorly understood, although metabolic-related defense [12] and roles of roots in plants toward herbivore attack have attracted increasing interest.

Metabolic-related defense and crosstalk between above- and below ground parts have been proved to be measures to defend or tolerate herbivorous insects for infested plants. It is well documented that herbivore would induce accumulation of some metabolites which are toxic, repellant or anti-digestive for herbivores in leaves and resources allocation to roots upon attack by leaf chewers. Some secondary metabolites have been confirmed to be defensive compounds including alkaloids, phenols, flavonoids, terpene, and acetones. Role of roots in conferring resistance or tolerance against herbivores is attracting great scientific interest recent years. Roots not only provide water and minerals to plants but also synthesize some secondary metabolites, serve as a dynamic storage organ and act as a sensor to perceives environmental factors [13]. Studies have shown that root confers herbivore resistance to plants by synthesizing some resistance-related metabolites such as nicotine and enable plants to tolerate above-ground herbivores by increasing their root nutrient pools [14], [15], [16], [17].

For the protective role of defensive compounds and roots for insect-infested plants, we suppose that plants exposed to HIPVs may reconfigure their metabolic profiling in both leaves and roots.

In order to unveil this hypothesis, seedlings of *Ammopiptanthus mongolicus* and larvae of *Orgyia ericae* were used as a model system in this experiment. Leaves and roots of control seedlings, seedlings infested with larvae of *O. ericae* and seedlings exposed to HIPVs induced by arvae of *O. ericae* feeding from infested seedlings were analyzed with non-targeted NMR-based metabolic technology and processed with multivariate data analysis. The results presented revealed that HIPVs exposure led to similar metabolic profiling in both leaves and roots as those induced by herbivore.

## Materials and Methods

### Plants and insect materials

Seeds of *Ammopiptanthus mongolicus* (Maxim.) Cheng f. and larvae of *Orgyia ericae* Germar (Lepidoptera: Lymantriidae) were approved by Forest Pest control and Quarantine Station of Inner Mongolia's Alxa League to obtain from Alxa Left Banner and conduce this experiment. Larvae of *O. ericae* are one kind of main pests for *A. mongolicus*. *A. mongolicus* is an endangered plant, and the aim of the studies permitted by Forest Pest control and Quarantine Station of Inner Mongolia's Alxa League for this plant is to protect it from negative impacts caused by herbivorous insects.

Mature and healthy seeds of *A. mongolicus* harvested in August, 2011 were selected to germinate and grow in growth chamber at 25±2°C, 60-65% relative humidity, with a L14: D10 photoperiod in pots filled with a mixture of perlite: vermiculite: coarse sand: loam = 2∶2∶3∶5. Forty five healthy seedlings with seven or eight fully expanded leaves were randomly selected for experiments. Larvae of *O.ericae* were arrested with the help of the Forest Pest control and Quarantine Station of Inner Mongolia's Alxa League from Alxa Left Banner, Inner Mongolia. All larvae were maintained in growth chamber with the same parameters with those for *A. mongolicus* seedlings.

Herbivore and HIPVs exposure experiment was conducted in two growth chambers with identical growth conditions. A total of 30 seedlings with 15 each for herbivore and HIPVs exposure treatments were placed in one growth chamber, and 15 additional seedlings used as control were placed in the second growth chamber. Seedlings for herbivore-challenge treatment were equally divided into three groups and placed randomly at the bottom layer of the growth chamber. Similarly for HIPVs exposure treatment, three groups of seedlings were placed randomly on the second layer of the growth chamber. In this experiment, seedlings on the bottom layer were infested with larvae of *O. ericae* whereas seedlings on the second layer in the same growth chamber were exposed to HIPVs elicited by *O. ericae* feeding from seedlings on the bottom under air circulation. Therefore infested plants on the bottom layer were used as source plants for HIPVs whereas intact seedlings on the second layer were used as receiver plants. Control seedlings were also divided into three equal groups and randomly placed on a layer located between the bottom and the second layer of the growth chamber.

Leaves except cotyledons of each seedling were covered with two transparent plastic culture plates (9 cm in diameter). For herbivore experiment, two 3rd instar *O. ericae* larvae were transferred into each pair of plates at 10:30 a.m. and removed at 6:30 p.m. for group one, 10:30 p.m. for group two and 10:30 a.m. on the next day for group three. Leaves and roots from these seedlings were collected immediately after removal of the larvae. Leaves and roots of receiver plants and control plants with the same group numbers with infested plants were also collected at the three time points respectively.

Small holes were made on the top of the culture plates to provide ventilation. For the passing of the seedling stems across the culture plates, gaps with appropriate sizes were made on the edges of these plates. Additionally, transparent tape was used to stick the plates for one seedlings together to prevent larvae from escaping or damaging other parts of the plants.

### Extraction of plant materials

Leaves and roots were extracted according to the method used by Kim *et al* [18] with some modifications as in our previous study [19]. Plant materials were grinded with pestle and mortar under liquid nitrogen into fine powder, and then were freeze-dried for 24 h. After weighting dried samples, solvent containing 0.75 ml CD_3_OD and 0.75 ml KH_2_PO_4_ buffer in D_2_O (pH 6.0) with 0.05% (wt/wt) TSP were added into each centrifuge tube containing dried powder of fifty milligram leaves or forty milligram roots. The centrifuge tubes were subsequently vortexed for 1 min and ultrasonicated for 20 min at 25°C. To get supernatant for NMR test, centrifuge tubes containing plant materials and extraction solvent were finally centrifuged for 15 min (13000 rpm, 4°C).

### Metabolic profiling

NMR spectra were recorded on a 600 MHz JEOL spectrometer operating at a proton frequency of 600.17 MHz at 25°C with D_2_O as the internal lock. All ^1^H NMR spectra were acquired with inverse detection probe. The parameters used were 32 sans, 32K points, spectral width of 15 ppm, 90° pulse length of 5.2 µs and relaxation delay of 15 s. To suppress the residual water signals, a pre-saturation sequence was used at the water frequency during the recycle delay. Parameters for 2D spectra were setted as those in previous studies [18]. ^1^H NMR spectra free induction decays were zero filled to 64K before Fourier transformation, and then were transformed with a line broadening factor of 0.3. Transformed ^1^H spectra were manually phased, baseline corrected and calibrated to TMSP at 0.0 ppm with Mnova (version 8.1.2, Bruker). 2D NMR spectra including ^1^H J-resolved spectroscopy (JRES), ^1^H-^1^H correlation spectroscopy (^1^H-^1^ HCOSY), ^1^H-^1^H total correlation spectroscopy (^1^H-^1^H TOCSY), heteronuclear single quantum coherence spectroscopy (HSQC) and heteronuclear multiple bond correlation (HMBC) were also recorded on the 600 MHz JEOL spectrometer with parameters described in previous research [18].

### Data analysis

With Mnova (version 8.1.2, Bruker), all ^1^H NMR spectra were binned with equal width of 0.04 ppm automatically over the region of 0.1–10.0 ppm for leaves and 0.4–10.0 ppm for roots. Spectral intensities were subsequently scaled to total area or intensity of TMSP. Binned data excluding the signals for residual water and methanol (4.8–4.9 ppm and 3.28–3.34 ppm) were then used for multi variate data analysis. multi variate data analysis was performed with RStiodio [20].

To assess metabolic changes induced by herbivore and HIPVs exposure in leaves and roots, principal component analysis (PCA) and orthogonal projections to latent structures discriminate analysis (OPLS-DA) were performed with RStudio to get grouping information, and identify significantly changed compounds. Data used for PCA were scaled with Pareto scaling method, whereas data for OPLS-DA were mean centered and scaled to unit variance. OPLS-DA was performed with the binned ^1^H NMR data as X-matrix and class information as Y-matrix [21]. For the statistical significance, a cut value of 0.811 (i.e., |r|>0.811) for correlation coefficients was used (*n* = 5, *P*<0.05) according to the discriminating significance of the Pearson's product-moment correlation coefficient [22]. Back transformed loadings of OPLS-DA were plotted against ^1^H chemical shift with square of coefficients (r^2^) as color codes. To get correlation coefficients of significantly changed metabolites, concentrations of metabolites significantly changed based on the results of OPLS-DA on binned spectra were calculate with signals which were not overlapped with other signals according to concentration of TMSP and applied with PCA and OPLS-DA.

The quality of all the models were judged by R^2^X (Fraction of Sum of Squares of all the X's explained by the components used in the model), Q^2^ (The fraction of the total variation of the Y's that can be predicted by the components used in the models) with cross-validation, and the validity of OPLS-DA models were further confirmed with 200 times permutation test [23].

## Results

### Metabolite identifications from NMR resonance

**Figure 1:**
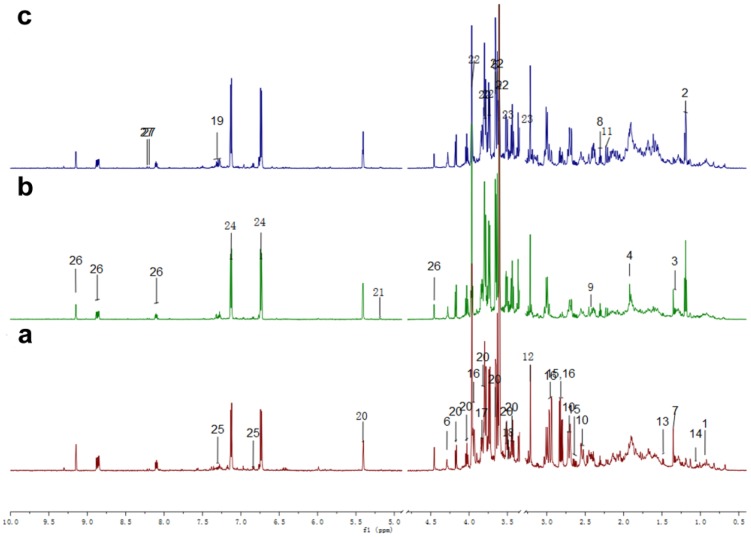
Typical ^1^H NMR spectra of leaves. a, ^1^H NMR spectra of leaves from control plants for 12 h; b, ^1^H NMR spectra of leaves from seedlings infested for 12 h; c, ^1^H NMR spectra of leaves from seedlings exposed to HIPVs for 12 h. 1: α-Linoleic acid analogues, 2: ethanol, 3: threonine, 4: acetic acid, 5: oxalacetic acid, 6: glycolate, 7: α-hydroxyisobutyric acid, 8: glutaric acid, 9: succinic acid, 10: citric acid, 11: acetone, 12: choline, 13: alanine, 14: valine, 15: aspartic acid, 16: asparagin, 17: serine, 18: glycine, 19: phenylalanine, 20: sucrose, 21: α-Glucose, 22: pinitol, 23: inositol, 24: hydroxybenzene derivative 1, 25: 4-hydroxyphenylacetic acid, 26: trigonelline, 27: adenine.

Metabolites in leaves were assigned by comparison with chemical shifts and constants of our previous results and confirmed by 2D NMR spectra including J-resolved spectra, COSY, TOCSY, HSQC and HMBC or pure compounds, and labeled with numbers on ^1^H NMR spectra (Figure 1). Metabolites in roots were identified according to our previous results [19] and literature data [18], [24], [25], 2D NMR spectra or pure compounds and the ^1^H chemical shifts and coupling constants of identified signals were listed in table 1. Metabolites identified from leaves and roots included fatty acids, organic acids, amino acids, sugars, alcohols, aromatic compounds, alkaloids and isoflavones.

**Table 1:** ^1^H chemical shifts and coupling constants (J, Hz) of metabolites in *A. mongolicus* roots identified (CD_3_OD-KH_2_PO_4_ in D_2_O, pH 6.0)

compounds	Chemical shifts (ppm) and coupling constants (Hz)
α-Linoleic acid analogues	0.96 (t, J = 7.4 Hz)
Ethanol	1.19 (t, J = 6.9 Hz)
	3.64
Threonine	1.33 (d, J = 6.9 Hz)
	4.04
	4.22
Acetic acid	1.94 (s)
Oxalacetic acid	3.65 (s)
Glycolate	4.29 (s)
Acetone	2.23 (s)
Choline	3.21 (s)
Citric acid	2.74(d, J = 17.3 Hz)
	2.53(d, J = 17.3 Hz)
Alanine	1.48 (d, J = 7.4)
	3.71
Valine	1.00 (d, J = 7.1 Hz)
	1.06(d, J = 6.9 Hz)
Aspartic acid	2.64 (dd, J_1_ = 17.4 Hz, J_2_ = 9.3 Hz)
	2.81(dd, J_1_ = 17.5 Hz, J_2_ = 3.3 Hz)
Asparagin	2.82 (dd, J_1_ = 17.1 Hz, J_2_ = 8.2 Hz)
	2.96 (dd, J_1_ = 17.0 Hz, J_2_ = 4.0 Hz)
Glycine	3.49(s)
Sucrose	5.40 (d, J = 3.6 Hz)
	4.17 (d, J = 8.9 Hz)
	4.03 (t, J = 8.3 Hz)
	3.762–3.862(m)
	3.73(dd, J_1_ = 10.2 Hz, J_2_ = 9.1 Hz)
	3.65 (s)
	3.51 (dd, J_1_ = 9.9 Hz, J_2_ = 3.8 Hz)
	3.43 (dd, J_1_ = J_2_ = 9.7 Hz)
Fructose	4.07(dd, J_1_ = 3.5 Hz, J_2_ = 1.5 Hz)
α-Glucose	5.19 (d, J = 3.5 Hz)
β-Glucose	4.58 (d, J = 7.9 Hz)
Pinitol	3.96 (m)
	3.73 (dd, J_1_ = 9.5 Hz, J_2_ = 9.5 Hz)
	3.77 (dd, J_1_ = 10.0 Hz,J_2_ = 2.8 Hz)
	3.60 (s)
	3.63 (dd,J_1_ = J_2_ = 9.6 Hz)
	3.32 (t, J = 9.4 Hz)
Hydroxybenzene derivative 1^a^	7.13 (d, J = 8.9 Hz)
	6.73 (d, J = 8.9 Hz)
Onion	8.33(s)
	8.31(s)
	8.18(d, J = 8.3 Hz)
	7.49(d, J = 9.8 Hz)
	7.31(d, J = 3.3 Hz)
	7.27(dd, J_1_ = 8.1, J_2_ = 3.0 Hz)
	7.07(d, J = 9.0)
	5.23(d, J = 8.1)
Aromatic compound 1^b^	7.46(d, J = 8.6 Hz)
	6.83(dd, J_1_ = 8.5 Hz, J_2_ = 2.4 Hz)
	6.64(d, J = 2.4 Hz)
	6.91(s)
	6.45(s)
Trigonelline	9.14 (t, J = 1.4 Hz)
	8.87 (ddd, J_1_ = 8.3 Hz, J_2_ = 2.3 Hz, J_3_ = 2.3 Hz)
	8.85 (ddd, J_1_ = 6.2 Hz, J_2_ = 2.1 Hz, J_3_ = 2.1 Hz)
	8.10 (dd, J_1_ = 7.9 Hz, J_2_ = 6.2 Hz)
	4.45 (s)

a Notes: signals can just be identified as hydroxybenzene derivatives;

b Notes: signals can just be identified as Aromatic compound.

### Similar metabolic profiling induced by herbivore and HIPVs

**Figure 2:**
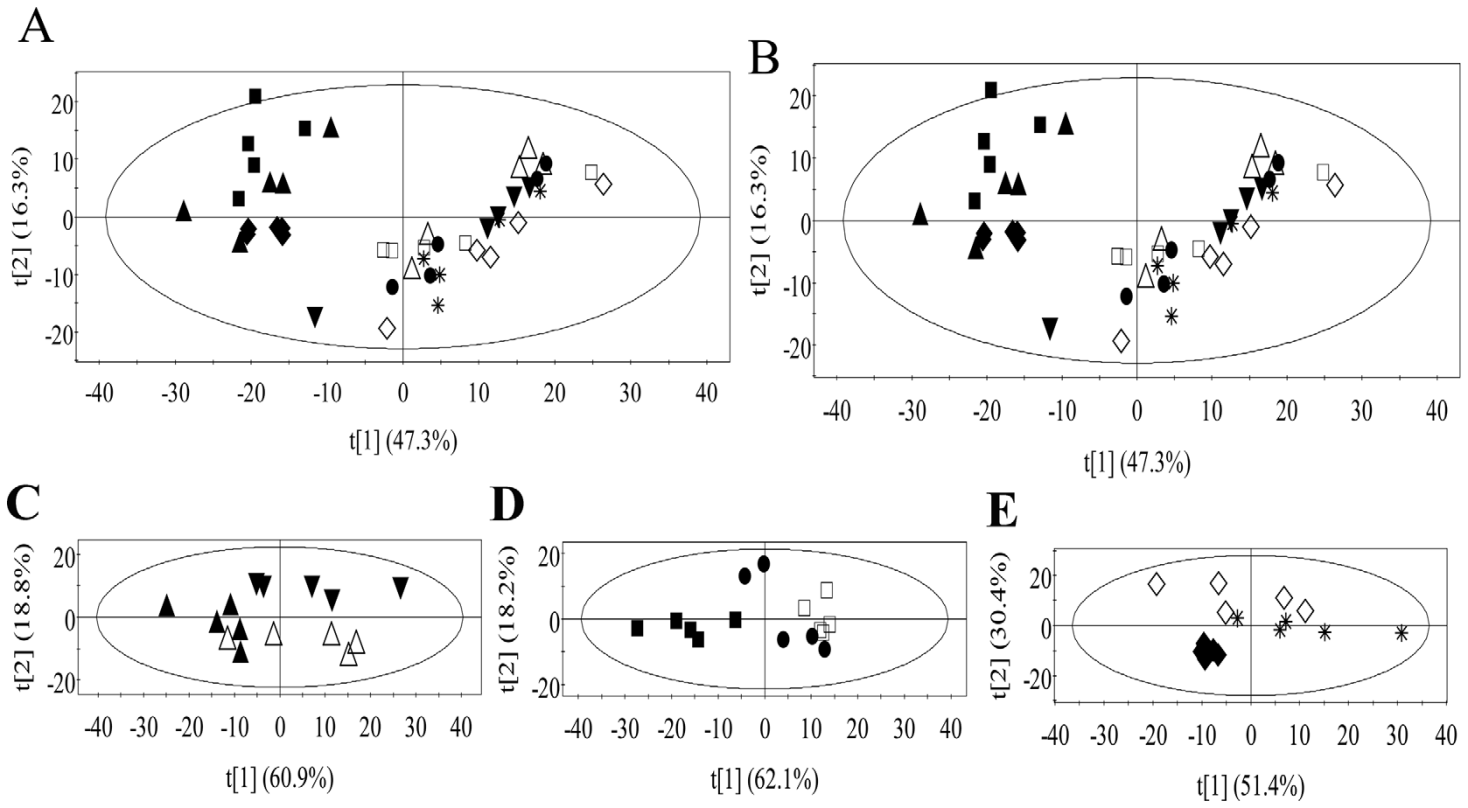
PCA score plots of leaves and roots. A, PCA score plot of all leaves obtained from binned ^1^H NMR data scaled to intensityof TMSP; B, PCA score plot of all leaves obtained from binned ^1^H NMR data scaled to total area of the corresponding spectra; C, PCA score plot of roots from control seedlings, infested seedlings and HIPVs induced seedlings at 6 h obtained from binned ^1^H NMR data scaled to intensity of TMSP; D, PCA score plot of roots from control seedlings, infested seedlings and HIPVs induced seedlings at 12 h obtained from binned ^1^H NMR data scaled to intensity of TMSP; E, PCA score plot of roots from control seedlings, infested seedlings and HIPVs induced seedlings at 24 h obtained from binned ^1^H NMR data scaled to intensity of TMSP; leaf samples were symbolized as follows: ▴, control for 6 h; ▪, control for 12 h; ♦, control for 24 h; △, leaves infested for 6 h; □, leaves infested for 12 h; ◊, leaves infested for 24 h; ▾, leaves exposed to HIPVs for 6 h; •, leaves exposed to HIPVs for 12 h; *, leaves exposed to HIPVs for 24 h. Symbolizations of root samples were corresponded with the symbols of leaves. Percents of the total variances for the evaluated samples explained by the first two principal components were labeled following t [1] and t [2] respectively.

PCA, an unsupervised reliable grouping method, was firstly applied to get grouping information for all leaves and roots. PCA score plot of leaves obtained from binned ^1^H NMR data scaled intensity of TMSP (Figure 2A) with first two principal components explaining 47.3% and 16.3% of the variances for all leaf samples showed that leaves infested or exposed to HIPVs form a group that separated clearly from those of control seedlings. Therefore, herbivore and HIPVs exposure induced similar metabolic profiling in *A. mongolicus* leaves which were considerably different from those of control and both infestation and HIPVs exposure did not cause further significant metabolic change after 6 h.

Leaves are food for leaf chewers to get enough and comprehensive nutrition to grow and develop, therefore, composition and proportions of metabolites in leaves reflect the quality of these leaves for these herbivores. To investigate the changes of relative content of all metabolites, data of binned ^1^H NMR spectra were scaled to the total area of the corresponding ^1^H NMR spectra. Interestingly, clear separation between leaves obtained from control seedlings and those from infested plants or HIPVs exposed plants were achieved whereas the later two kinds of leaves grouped together regardless of harvest time in score plot of PCA (Figure 2B). Therefore, relative contents of metabolites in leaves infested with larvae of *O. ericae* and leaves exposed to HIPVs were similar and they were dramatically different from those in leaves of control seedlings and no further metabolic change was induced by herbivore and HIPVs exposure after 6 h.

For roots, PCA were applied on binned data from ^1^H NMR spectra scaled to concentration of TMSP according to harvest time. As shown in PCA score plots, (Figure 2C–E) roots from seedlings exposed to HIPVs for 6 h separated from control and roots from infested seedlings, whereas the later two kinds of roots did not separate clearly. At both 12 h and 24 h, generally two groups of samples were observed and roots from control seedlings grouped alone away from roots from seedlings attacked by herbivore or exposed to HIPVs. Roots of *A. mongolicus*, therefore, responded similarly at metabolic level to larvae of *O. ericae* attack and HIPVs exposure at 12 h and 24 h but not for 6 h.

To confirm the similarity of metabolic response of *A. mongolicus* to *O. ericae* larvae attack and HIPVs exposure, OPLS-DA models with 200 times permutation test were applied on the pairwise comparisons of leaves or roots from infested seedlings or HIPVs induced seedlings according to harvest time. No significant metabolic change was observed for these leaves or roots.

### Metabolic changes in leaves induced by HIPVs and herbivore

To follow approach the detailed metabolic changes and identify differences in significantly changed metabolites induced by herbivore and HIPVs exposure, OPLS-DA was applied to the binned ^1^H NMR data scaled to concentration of TSP of challenged leaves versus control. Pairwise comparisons of leaves attacked by herbivore or exposed to HIPVs versus control were made over time. Permutation test of 200 times, R^2^X and Q^2^ indicated that both herbivore attack and exposure to HIPVs induced significant metabolic changes at all three treated time periods.

To get more accurate correlation relationship of significantly changed metabolites, concentrations of significantly changed metabolites identified from the results of OPLS-DA of binned ^1^H NMR data were calculated according to the concentration of TMSP and further evaluated by OPLS-DA. Correlation coefficients of these metabolites were listed in table 2. Values of correlation coefficients in table 2 showed generally increase of ethanol and glycolate and decrase of other metabolites, however, relationship of specific metabolites were different with the integrated metabolic changes induced by herbivore and HIPVs exposure. Therefore, herbivore and HIPVs exposure induced similar but specific metabolic changes to *A. mongolicus* leaves.

**Table 2:** Correlation coefficients of significantly changed metabolites induced by herbivore or HIPVs exposure.

Metabolites (ppm)	Leaves infested with larvae of *O. ericae*			Leaves exposed to HIPVs		
	6 h	12 h	24 h	6 h	12 h	24 h
**Sugars**						
Sucrose 5.40	0.61	−0.63	0.75	0.82	0.51	0.64
**Organic acids**						
Succinic acid 2.42	−0.40	−0.00	−0.18	−0.69	−0.85	−0.64
Glutaric acid 2.30	−0.73	−0.04	−0.61	−0.11	−0.82	−0.95
Glycolate 4.29	0.93	0.94	0.99	0.98	0.94	0.99
**Amino acids**						
Valine 1.05	−0.92	−0.40	−0.90	−0.84	−0.87	−0.94
Aspartic acid 2.63	−0.94	−0.69	−0.90	−0.83	−0.89	−0.92
Asparagin 2.81	−0.91	−0.89	−0.96	−0.90	−0.56	−0.80
Threonine 1.33	−0.96	−0.68	−0.86	−0.95	−0.97	−0.78
Alanine 1.48	−0.90	−0.57	−0.87	−0.84	−0.68	−0.93
**others**						
α-Linoleic acid analogues 0.96	−0.86	0.60	−0.66	−0.79	−0.81	−0.76
Ethanol 1.19	0.94	0.96	0.99	0.96	0.98	0.95
Choline 3.21	−0.47	−0.20	−0.88	−0.08	−0.60	−0.94
Pinitol 3.73	−0.61	−0.90	0.13	0.11	−0.80	−0.49
Hydroxybenzene derivatives 1 7.13	0.11	0.26	−0.08	0.60	0.22	−0.29
Trigonelline 9.14	−0.91	−0.94	−0.95	−0.96	−0.96	−0.99
Adenine 8.20	−0.74	0.13	−0.74	−0.40	−0.75	−0.65

Positive value, increase; negative value, decrease; cut value for significance: 0.811.

### Metabolic changes in roots induced by herbivore and HIPVs exposure

**Figure 3:**
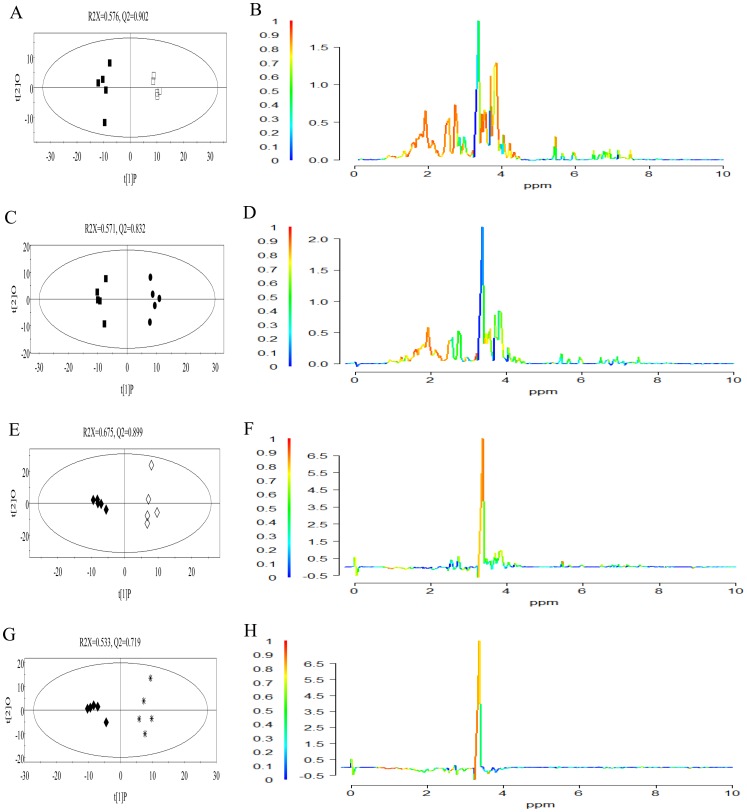
OPLS-DA score plots (left) and corresponding loading plots (right) of roots. A, B: OPLS-DA score and loading plots of roots from seedlings infested for 12 h versus control; C, D: OPLS-DA score and loading plots of roots from seedlings exposed to HIPVs for 12 h versus control; E, F: OPLS-DA score and loading plots of roots from seedlings infested for 24 h versus control; G, H: OPLS-DA score and loading plots of roots from seedlings exposed to HIPVs for 24 h versus control. Symbolization of samples in score plots were the same with figure 2. For coefficient-coded loading plots, horizontal axis corresponds to the integrated regions of 0.4–10.0 ppm in ^1^H NMR spectra; vertical axis are the back transformed loading value and colors for variables (compounds) are coded by square of coefficients (r^2^).

For the documented abilities to perceive signals from damaged leaves, biosynthesize of some secondary metabolites for leaves to defend herbivorous insects and enhance sink strength upon herbivore attack of roots, metabolic changes of roots were measured and analyzed with OPLS-DA. Results of OPLS-DA indicated that herbivore feeding and HIPVs exposure caused significant metabolic changes in roots compared with control at 12 h (Figure 3A, C) and 24 h (Figure 3E, G) but not for 6 h.

**Figure 4:**
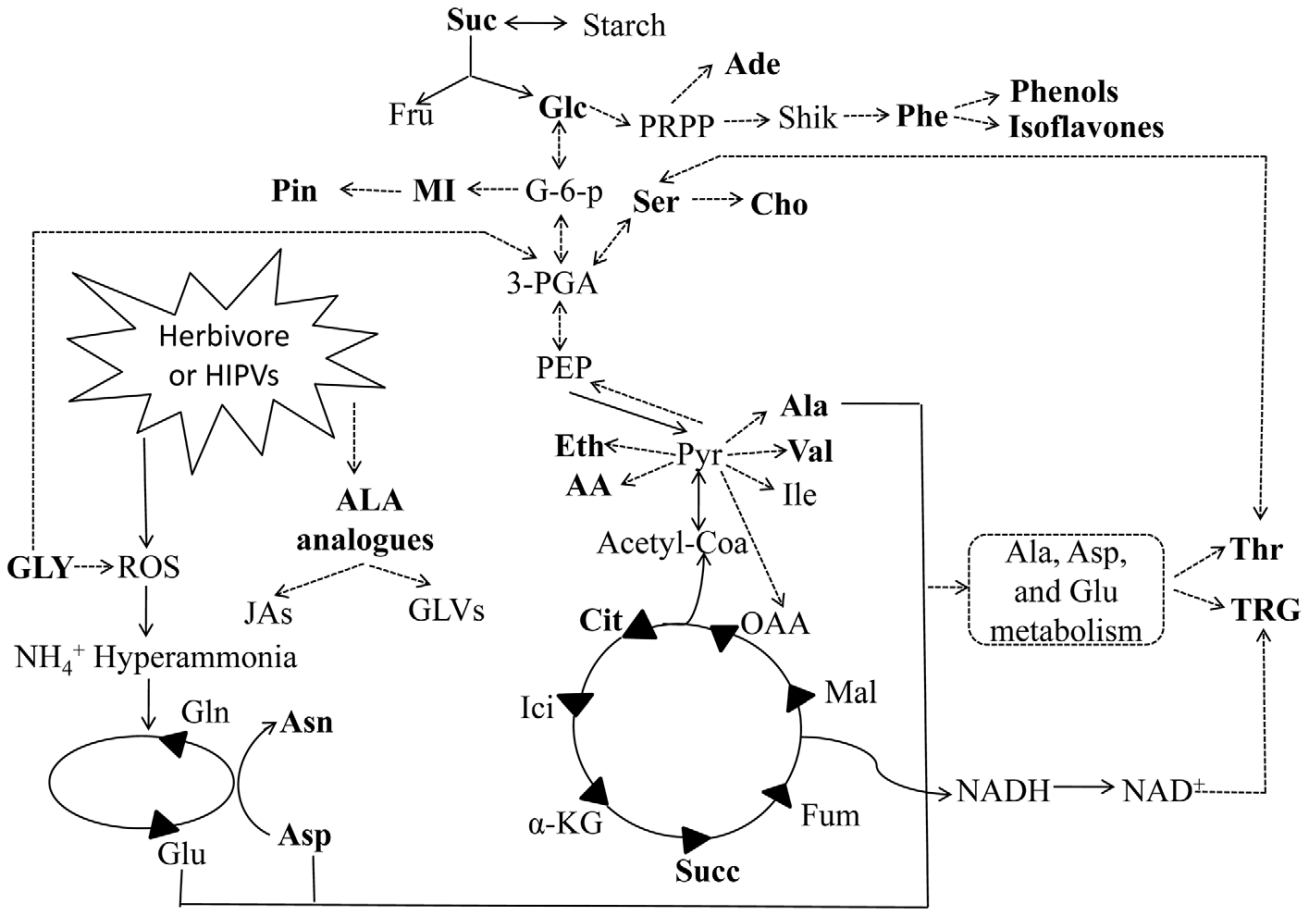
Herbivore and HIPVs exposure induced metabolic changes in leaves or roots. Metabolites identified were shown in bold letters. Dashed lines: multiple-step reactions; solid lines: one-step reactions. Abbreviations for metabolites: AA, acetic acid; Ade, adenine; Ala, alanine; ALA, α-linoleic acid analogues; Asn, asparagines; Asp, aspartic acid; Cho, choline; Cit, citrate; Eth, ethanol; F-6-P, fructose-6-phosphate; Fru, fructose; Fum, fumarate; Glc, glucose; Gln, glutamine; Glu, glutamate; GLVs, green leaf volatiles; GLY, glycolate; Ici, isocitrate; Ile, isoleucine; JAs, Jasmonates Mal, malate; MI, myo-Inositol; NAD^+^, nicotinamide adenine dinucleotide; NADH, nicotinamide adenine dinucleotide; OAA, oxaloacetate; PEP, phosphoenolpyruvate; 3-PGA, 3-phosphoglycerate; Phe, phenylalanine; Pin, pinitol; PRPP, 5-phosphoribosyl 1-pyrophosphate; Pyr, pyruvate; Ser, serine; Shik, shikimate; Suc, sucrose; Succ, succinate Thr, threonine; TRG, trigonelline; Val,valine; α-KG, alpha-ketoglutarate.

Loading plots indicated that roots from infested seedlings or HIPVs exposed seedlings accumulated higher level of some metabolites without decrease of any metabolite after treated for 12 h (Figure 3B, D). At 12 h, both roots from infested plants or plants exposed to HIPVs were characterized by higher level of valine, choline, acetic acid, serine, sucrose, succinic acid, alanine, asparagin, glutaric acid, threonine, acetone, 4-hydroxyphenylacetic acid, ethanol and some unidentified lower content organic acids, nitrogenous compounds (Figure 3B, D). However, roots from plants infested for 12 h also showed increased level of glycolate, pinitol, trigonelline, α-linoleic acid analogues, aspartic acid, citric acid phenylalanine and some unidentified sugars (Figure 3B), whereas extra increased metabolites in roots from plants exposed to HIPVs for 12 h included α-glucose, and adenine (Figure 3D) when compared with control. Additionally, higher levels and higher contributions for the increased compounds for the discrimination from control were observed in roots of infested seedlings compared with those of seedlings exposed to HIPVs for the hotter colors of color-coded loading plot in figure 4B and 4D.

For roots from seedlings infested or exposed to HIPVs for 24 h, decreased compounds included α-linoleic acid analogues, trigonelline, choline, threonine, ethanol, valine and some unidentified organic acids (Figure 3F, H) as well as specifically decrease of asparagin and unidentified compounds with double bounds (Figure 3F) for roots from infested seedlings and aspartic acid, succinic acid, alanine and hydroxybenzene derivatives 1, glutaric acid and unidentified nitrogenous compounds for roots from seedlings exposed to HIPVs (Figure 3H). No metabolite was observed to increase in roots exposed to HIPVs for 24 h (Figure 3H) whereas pinitol, sucrose and serine showed higher levels in roots from seedlings infested for 24 h (Figure 3F).

Larvae of *O. ericae* attack and HIPVs exposure, therefore, induced similar metabolic profiling but differently affected metabolic pathways in roots of *A. mongolicus*. Increase of sucrose and some amino acids and other small molecular compounds without decrease of metabolites after treated for 12 h may indicate resources allocation to roots from leaves induced by herbivore and HIPVs exposure for sucrose and amino acids could be transported from leaves to roots.

## Discussion

Over the last ten years, NMR-based metabolic technology has attracted significant interest in plant science primarily because of the minimal sample preparation, ease of quantitative analysis, detection not limited by ionization efficiency and chromophore structure, as well as the definitive structural information derived from crude extracts despite of its low sensitivity [18], [26], [27]. This technology has contributed immensely to understanding the effects on plants induced by biotic [28], [29], [30], [31], [32], [33], [34] or abiotic stresses [35], [36], [37], analyzing the metabolic difference in mutant [38] or transgenic plants [39], [40], monitoring specific metabolic pathway, discovering bioactive metabolites [41], [42], and classifying plant species [43], [44].

In this experiment, NMR-based metabolic technology combined with multi variate data analysis revealed similar but specific metabolic changes induced by HIPVs exposure with those resulted from herbivore in both leaves and roots. Although defensive characterizations of plants exposed to HIPVs have been observed, this is the first time to reveal similar metabolic profiling induced by HIPVs exposure compared with those caused by herbivore. For metabolism reflect the results of gene expression and regulation of signal transduction, the metabolic changes resulted from HIPVs exposure may be the reason to reduced the development of larval [11].

Further data analyze with OPLD-DA revealed the differences in quality and quantity of metabolic changes in HIPVs exposed plants as compared to infested plants. Correlation coefficients in table 2 and loading plots of OPLS-DA (Figure 3B, D, F) showed that significantly changed compounds or changed levels induced by HIPVs exposure in leaves and roots at specific treat periods were different from those induced by herbivore. HIPVs, therefore, may activate part of defensive response in *A. mongolicus* which may be related with defense cost for these plants were still not attacked by herbivore.

HIPVs exposure affected primary and secondary metabolisms of leaves and roots of *A. mongolicus* similarly with those induced by herbivore. For leaves and roots, gluconeogenesis/glycolysis, TCA cycle, glycolate pathway, metabolim of amino acids and lipids, and secondary metabolisms were affected (figure 4). Increase of glycolate in leaves may indicate activation of glycolate oxidase which is related with production of H_2_O_2_ in plant cells upon herbivore attack as reviewed in the signaling plot [45]. Increased ethanol may be the product of anaerobic respiration caused by higher concentration of CO_2_ within cells produced by enhanced glycolate pathway. Higher level of phenylalanine in roots from seedlings infested for 12 h may indicate activation of phenylpropanoids pathway which links primary metabolism and secondary metabolism to biosynthesize coumarins, lingans and flavonoids [46]. For the multifunction of trigonelline, sun as promoting G2 arrest, biological role of DNA methylation, serving as inducer of defensive metabolism to oxidative stress, involving in circadian rhythm [47], role of increased trigonelline in root of *A. mongolicus* remained further research. Quinolizidine alkaloids are documented to be contained in *A. mongolicus* [48], [49] and are implicated in conferring herbivore resistance to plants. Reasons for not identifying these compounds may due to the small amounts in leaves and severe signal overlapping between 1.0 to 2.5 ppm. However, incease of nitrogenous compounds in roots after treated for 12 h or 24 h may indicate biosynthesis of these compounds induced by herbivore or HIPVs exposure. Low concentrations of isoflavone glucosides in *A. mogolicus* may also be one reason to the not significant changes for these compounds which is a class of herbivore-resistant compounds in other plants.

In addition to metabolic changes, decrease of several kinds of amino acids (such as valine, alanine, asparagin, threonine, aspartic acid) and some other small molecular compounds in leaves together with increase of these metabolites in roots at 12 h indicated resource reallocation induced by HIPVs exposure and herbivore. Roots are observed to enhance sink strength upon herbivore attack which is referred to as a strategy of herbivore tolerance. Fluxes of carbon and nitrogen have been observed from leaves to roots with radioisotopes in plant upon foraging herbivore attack such as sugars allocation to roots in *Nicotiana attenuata* damaged by simulated herbivore [16], more rapid photosynthate export rate from leaves to stem and roots of *Populus* after JA treatment [15], increased nitrogen (^13^N) export from leaves to roots in tomato elicited by methyl jasmonate [50]. Resource reallocation from damaged site to non-infested parts of plant is referred to as saving neutrinos for compensatory growth.

In conclusion, this study revealed that HIPVs released from *A. mongolicus* attacked by larvae of *O. ericae* induced similar but specific metabolic changes in yet-damaged conspecific seedlings in both leaves and roots.
